# Effect of the Combination of Synthetic Anthelmintics with Carvacryl Acetate in Emulsions with and without a Sodium Alginate Matrix on *Haemonchus contortus*

**DOI:** 10.3390/ani14071007

**Published:** 2024-03-26

**Authors:** Livia Furtado Ximenes, Henety Nascimento Pinheiro, José Vilemar de Araújo Filho, Weibson Paz Pinheiro André, Flávia Oliveira Monteiro da Silva Abreu, Mayrla Rocha Lima Cardial, Debora de Souza Colares Maia Castelo-Branco, Ana Carolina Fonseca Lindoso Melo, Francisco Flávio da Silva Lopes, Selene Maia de Morais, Lorena Mayana Beserra de Oliveira, Claudia Maria Leal Bevilaqua

**Affiliations:** 1 Laboratório de Doenças Parasitárias, Programa de Pós-Graduação em Ciências Veterinárias, Faculdade de Veterinária, Universidade Estadual do Ceará, Fortaleza 60714-903, Ceará, Brazil; liviafx.nib@gmail.com (L.F.X.); vilemar_filho@hotmail.com (J.V.d.A.F.); weibsonpaz@hotmail.com (W.P.P.A.); lorena.mayana@uece.br (L.M.B.d.O.); 2 Laboratório de Química Analítica e Ambiental, Programa de Pós-Graduação em Ciências Naturais, Universidade Estadual do Ceará, Fortaleza 60714-903, Ceará, Brazil; henety.pinheiro@aluno.uece.br (H.N.P.); flavia.monteiro@uece.br (F.O.M.d.S.A.); mayrla.lima@uece.br (M.R.L.C.); 3 Departamento de Patologia e Medicina Legal, Faculdade de Medicina, Universidade Federal do Ceará, Fortaleza 60714-903, Ceará, Brazil; deb_castelobranco@yahoo.com (D.d.S.C.M.C.-B.); acflmelo@gmail.com (A.C.F.L.M.); 4 Laboratório de Química de Produtos Naturais, Programa de Pós-Graduação em Ciências Veterinárias, Faculdade de Veterinária, Universidade Estadual do Ceará, Fortaleza 60714-903, Ceará, Brazil; flaviollopez@gmail.com (F.F.d.S.L.); selene.morais@uece.br (S.M.d.M.)

**Keywords:** levamisole, ivermectin, thiabendazole, synergism

## Abstract

**Simple Summary:**

One of the biggest problems with sheep and goat farming is worms. These parasites impede the development of these animals and even lead to death. Currently, the dewormers used to control these parasites do not have the desired effect because worms have developed resistance. This study aimed to evaluate the effect of nanoemulsions that combine synthetic anthelmintics with carvacryl acetate on the worm *Haemonchus contortus*. Assessments were performed on the hatching of worm eggs and the development of larvae. Photomicrographs of the eggs were taken to show the effect of the different treatments. The best results were obtained with the combination of carvacryl acetate and thiabendazole and carvacryl acetate and thiabendazole encapsulated in sodium alginate. The results of this study allowed the reuse of anthelmintics such as benzimidazoles whose use was discontinued due to the development of anthelmintic resistance, when combined with natural and nanoencapsulated products.

**Abstract:**

The present study aimed to evaluate the effect of nanoemulsions using combined synthetic anthelmintics, thiabendazole (TBZ), levamisole (LEV), and ivermectin (IVM), with carvacryl acetate (CA) against *Haemonchus contortus*, and also tested the presence and absence of alginate (ALG). The anthelmintic effect of the CA/TBZ nanoemulsion was evaluated in the egg hatch test (EHT). The effects of CA/IVM and CA/LEV nanoemulsions were evaluated in the larval development test (LDT). The emulsions CA/TBZ/ALG and CA/TBZ showed a multimodal profile, with most particles on the nanometric scale. The encapsulation efficiency in CA/TBZ/ALG was 80.25%, and that in CA/LEV/ALG was 89.73%. In the EHT, CA/TBZ and CA/TBZ/ALG showed mean combination indices (CIs) of 0.55 and 0.36, respectively, demonstrating synergism in both. In LDT, CA/IVM had an average CI of 0.75, and CA/LEV and CA/LEV/ALG showed CI values of 0.4 and 0.93, respectively. It was concluded that CA/TBZ showed a synergistic interaction, and CA/TBZ/ALG showed an enhanced effect. In addition, the matrix brought stability to the product, encouraging its improvement to obtain higher efficacy.

## 1. Introduction

Parasitism by gastrointestinal nematodes (GINs) is socioeconomically important due to the cost of anthelmintics, the drop in production, and animal deaths. Despite significant advances in the treatment of these diseases in the middle of the last century, they remain a threat to livestock. Effective control of GINs is therefore essential for the profitability of livestock production, depending heavily on the routine use of synthetic drugs. However, this approach is unsustainable, as resistance to anthelmintic drugs is widespread and growing [1].

The term “drug combination” refers to formulations that contain two or more classes of drugs with a similar spectrum of activity but different modes of action that are commonly used for chemotherapeutic indications in human medicine, such as cancer, as well as viral, bacterial, and protozoal infections. The use of these combinations can improve the effectiveness of treatment in the presence of resistance to anthelmintics, as demonstrated for several pathogens, based on the knowledge gained from the use of insecticides, pesticides, and herbicides [2,3,4].

The combination of synthetic anthelmintics with secondary metabolites isolated from essential oils may be an alternative for the control of GINs, as there are already promising results in the control of fungi, bacteria, and ticks using the combination of synthetic and natural compounds [5,6]. In vitro studies found that the combination of bioactive compounds with albendazole and ivermectin was effective in inhibiting the hatching of *Haemonchus contortus* eggs [7]. A bioactive compound that showed promising anthelmintic action was carvacrol, a phenolic monoterpene isolated from essential oils that, after acetylation, showed promising anthelmintic efficacy on GINs of small ruminants and less toxicity than carvacrol [8].

Nanoemulsions are defined as kinetically stable systems whose droplets generally have a size range of 10 to 1000 nm [9]. They have long-term physical stability, with no visible flocculation or coalescence for long periods. This stability can be attributed to the impact of steric stabilization that occurs when using nonionic surfactants or polymers [10]. They also have other advantages, such as the smaller amount of surfactant needed compared to microemulsions, the uniform surface coating, good wetting, and spreading penetration capacity [11], and can improve the effectiveness of the active components [12]. Alginate is a biodegradable and biocompatible copolymer of guluronic acid and mannuronic acid that exhibits biopharmaceutical properties, such as pH sensitivity, biocompatibility, biodegradability, mucus adhesiveness, no toxicity, and no immunogenicity, and is an attractive excipient for the modified release of drugs. Sodium alginate nanoparticles were shown to improve the potential carriers of chemotherapeutic drugs [13]. Thus, the combination of synthetic anthelmintics with bioactive compounds could be an alternative to delay the selection of resistant populations, and nanotechnology can be a way to improve the stability and enhance the effect of active compounds.

## 2. Materials and Methods

### 2.1. Acetylation of Carvacrol

One gram of carvacrol (Sigma-Aldrich^®^ St. Louis, MO, USA—W224502) was added to acetic anhydride (15 mL) and sodium acetate (1.5 g). Acetic anhydride acted as an acetylating agent, and sodium acetate acted as a catalyst. The mixture was refluxed for 1 h. The room temperature solution was added to water (20 mL) and neutralized to pH 7.0 with 5% sodium bicarbonate. The reaction mixture was transferred to a separatory funnel and washed three times with chloroform (100 mL). The chloroform layer containing acetylated material was washed with water and then dried over sodium sulfate. The solvent was evaporated [14]. The product was subjected to thin-layer chromatography and characterized by infrared spectroscopy (FTIR) (Model 8300 Shimadzu Corporation, Kyoto, Japan).

### 2.2. Preparation of Emulsions

Four emulsions (O/W) were prepared (Table 1) by the means of a high-speed homogenization method, as described by [15], by studying the influence of the type of synthetic anthelmintic (TBZ or LEV) combined with CA and the presence of ALG as an external coating on the stability and related properties of the nanoemulsions. First, Tween 80 was added to CA/TBZ or CA/LEV and then subjected to an ultrasonic bath for 5 min, forming the oil phase. The oil phase was added to the distilled water or to an ALG 1% solution to form the nanoemulsions with the aid of a mechanical homogenizer Model ULTRA380 (Ultra stirrer) at 15,000 rpm and at 18,000 rpm, respectively, for CA/TBZ and CA/LEV emulsions over 2 min. Emulsions containing only water and Tween 80 (TBZ, IVM, or LEV) combined with CA were sonicated in a state-of-the-art sonicator (Fisher Scientific, Waltham, MA, USA) for 6 min in the range of 40.

### 2.3. Physicochemical Characterization of Nanoemulsions

A study was carried out on the physical stability of the emulsion from 24 h to 30 days. For this study, 10 mL of each emulsion was placed in a closed bottle, protected from light, and kept at a temperature of 26 °C. Periodic observations were carried out to detect any visual signs of instability, such as the formation of creaming, sedimentation, or the separation of phases.

### 2.4. Homogeneity and Morphology

An inverted microscope was used to observe the homogeneity and morphology of the dispersion. One drop of each formulation was placed on a glass microscope slide and covered with a coverslip. Emulsions were analyzed at 40× magnification.

### 2.5. Particle Size and Zeta Potential

To identify the diameter of the particles, the experiments were carried out with Malvern Zetasizer/Nanoseries Z590 equipment analytic (Panalytical Ltd., Malvern, UK). Solutions were made at 1% (*v*/*v*) concentration of the emulsions diluted with distilled water without magnetic stirring. One milliliter of these solutions was placed in a suitable cuvette to determine their particle diameters.

### 2.6. Infrared Spectroscopy Analysis

The detection of the main vibrational stretching modes was carried out by means of spectroscopy in the infrared region by FTIR. Sample readings were performed on a Shimadzu spectrometer from 400 to 4000 cm^−1^ using KBr inserts.

### 2.7. Encapsulation Efficiency

The active ingredient contained in the studied combinations, CA/TBZ and CA/LEV, in the emulsions was determined by dissolving 2 mL of each formulation in P.A. ethanol (10 mL). The incorporated active contents were determined by UV–Vis absorption at 290 nm and calculated using a calibration curve of each blend (mixture) of active principles in the solvent to determine the relationship between absorbance and concentration, as explained in Equations (1) and (2), respectively.

For the combination CA/TBZ, the relationship between the instrumental response (absorbance) and the concentration of the system is found in Equation (1):

(1) $$y=2.4525\;x+0.0679\;\;\;R^{2^{\prime }}=0.970$$

For the combination of CA/LEV, the relationship between the instrumental response and the concentration of the system is found in Equation (2):

(2) $$y=1.398\;x+0.0047\;\;\;R^{2^{\prime }}=0.988$$

Following analysis of the formulations, each emulsion (CA/TBZ and CA/LEV, individually) was diluted with ethanol (2 mL of emulsion to 10 mL of solution, ratio 1:4) and vigorously stirred for 10 min. Each emulsion had its encapsulation efficiency (%*EE*) determined in duplicate. The %*EE* calculation was obtained through Equation (3) [16].

(3) $$EE\;\left(\%\right)=\frac{m_{d}}{m_{T}}\times 100$$

*m_d_* = mass of combination (CA/TBZ or CA/LEV) determined by the calibration curves.

*m_T_* = total mass of composition (CA/TBZ or CA/LEV) added in the emulsion formulation.

### 2.8. In Vitro Release Profile

For this analysis, it was necessary to construct calibration curves for the individual formulations (CA/TBZ and CA/LEV) to calculate the concentrations released at time intervals as a function of absorbance (UV–Vis at 296 nm) using a solution of 1% Tween 80 (*v/v*) in water as the solvent. Only the CA/TBZ emulsion presented the possible readings for the construction of the curve. The calibration curve for CA/TBZ in 1% Tween 80 is shown in Equation (4).

(4) $$y=2.6341x+0.2193\;\;\;R^{2^{\prime }}=0.991$$

### 2.9. Scanning Electron Micrography

The morphological characterization of the emulsions was performed by scanning electron microscopy (SEM), using an accelerating voltage of 20 kV and a magnification of 5000 times. The samples were pre-coated with platinum using a cathodic spray applicator.

### 2.10. Haemonchus contortus Isolate

The Kokstad isolate (KOK) of *H. contortus* was used as a reference for being resistant to benzimidazoles, levamisole, and macrocyclic lactones [17,18]. The isolate was supplied by the Institut National de Recherche pour l’Agriculture, l’Alimentation, et l’Environnement (INRAE).

### 2.11. Recovery of Eggs and Larvae of Haemonchus contortus

Two lambs were housed in metabolic cages and dewormed with 5 mg/kg LEV (Ripercol^®^), 0.2 mg/kg IVM (Ivomec^®^), and 2.5 mg/kg monepantel (Zolvix^®^) at a single dose every other day. After the total elimination of the natural infection, as confirmed by fecal egg count (epg) and coproculture, one of the animals was monospecifically infected with 5000 L3 of KOK isolated from *H. contortus*. This animal was used as a source of *H. contortus* eggs for the egg hatch test. The other animal was kept free of GIN and provided feces for the larval development test.

### 2.12. Proportions of Substances Used in the Combinations

Preliminary tests were carried out for each substance alone: TBZ, LEV, IVM, and CA. The results of these tests were used to calculate effective concentration values to inhibit 50% (EC_50_) of egg hatching and larval development. These EC_50_ values were used to determine the proportions of each substance in the drug combination.

### 2.13. Egg Hatch Test (EHT)

Feces were collected directly from the rectal ampoules of sheep experimentally infected with *H. contortus.* Eggs were retrieved according to [19]. To perform the EHT, 250 μL suspensions containing approximately 100 fresh eggs were incubated with the same volume of TBZ solution (T8904—Sigma-Aldrich^®^) in concentrations ranging from 0.05 to 0.003 mg/mL and CA in concentrations that ranged from 8 to 0.5 mg/mL diluted with 3% Tween 80 in distilled water. The combination CA/TBZ was evaluated at concentrations of 0.011 mg/mL TBZ + 0.653 mg/mL CA to 6 × 10^−4^ mg/mL TBZ + 0.04 mg/mL CA. In the negative control, 3% Tween 80 was used. After incubation for 48 h, two drops of Lugol’s solution were added to the tubes, and then, eggs and first-stage larvae (L1) were counted under an optical microscope. Each trial was performed with five replicates and three repetitions [20].

### 2.14. Larval Development Test (LDT)

*Haemonchus contortus* eggs were incubated for 24 h to obtain L1. Solutions consisting of 1 mL of L1 (~250 larvae) and 1 mL of treatment solution diluted with 3% Tween 80 and distilled water were incubated with GIN-free sheep feces for 6 days at room temperature (27 °C) [21]. LEV concentrations (T1512—Sigma-Aldrich^®^) ranged from 0.08 to 0.005 mg/mL, IVM concentrations (I8898—Sigma-Aldrich^®^) ranged from 0.05 to 0.0003 mg/mL, and CA from 2 at 0.125 mg/mL. The combination CA/LEV was evaluated at concentrations of 0.014 mg/mL LEV + 0.383 mg/mL AC to 8 × 10^−4^ mg/mL LEV + 0.023 mg/mL AC; for the combination CA/IVM, the concentrations were from 8.4 × 10^−4^ mg/mL IVM + 0.383 mg/mL CA to 5 × 10^−5^ mg/mL IVM + 0.023 mg/mL CA. In the negative control, 3% Tween 80 was used. L3 were retrieved according to the technique described by [22] and counted under the microscope. Three replicates were performed with five repetitions for each treatment and the control.

### 2.15. Confocal Laser Scanning Microscopy (CLSM)

*Haemochus contortus* eggs exposed to CA (8 mg/mL), TBZ (0.05 mg/mL), CA/TBZ (0.011 mg/mL TBZ + 0.653 mg/mL CA), and CA/TBZ/ALG (0.011 mg/mL of TBZ + 0.653 mg/mL of CA) in the EHT were submitted to CLSM using the methodology previously described [23]. Tween 80 3% and ALG 1% were used as negative controls, and the emulsions were left under refrigeration for 48 h so that the eggs did not hatch. Eggs were placed between slides containing Live/Dead fluorescent dyes (Invitrogen^TM^, Waltham, MA, USA) and calcofluor. Then, the specimens were evaluated using a Nikon C2 Confocal Microscope (Nikon, Tokyo, Japan) at 40× magnification. Three lasers were used: 488 nm for the detection of the fluorescent dye SYTO9, which identifies live cells; 561 nm for the detection of propidium iodide, which identifies dead cells; and the third for the detection of calcofluor. 

## 3. Data Analysis

The egg hatch percentage was determined according to the following formula: (number of hatched larvae/number of hatched larvae + number of eggs) × 100.

The EC_50_ of synthetic anthelmintics, CA, and the combinations were calculated using the statistical program SPSS 8.0, version for Windows. Test results were analyzed by analysis of variance (ANOVA) followed by Tukey’s test. Version 1.0 of CompuSyn [24] was used for combination analysis. The effects of the combinations proposed by [25] were classified according to the combination index (CI) as strong synergism (CI < 0.1); synergism (0.1 > CI < 0.9); additive effect (0.9 > CI < 1.1); antagonism (1.1 > CI < 10); and strong antagonism (CI > 10). SRs between 0 and 10 were considered to signify slight synergism, and SRs *>* 10 were considered to signify high synergism [26].

## 4. Results

The emulsion CA/TBZ/ALG was evaluated for up to 45 days, during which time there was no macroscopic presence of any physical instability phenomenon, such as flocculation, sedimentation, and creaming. CA/LEV, CA/TBZ, and CA/LEV/ALG emulsions were evaluated for 30 days and did not show any signs of instability phenomena in the dispersion system.

**Figure 1:**
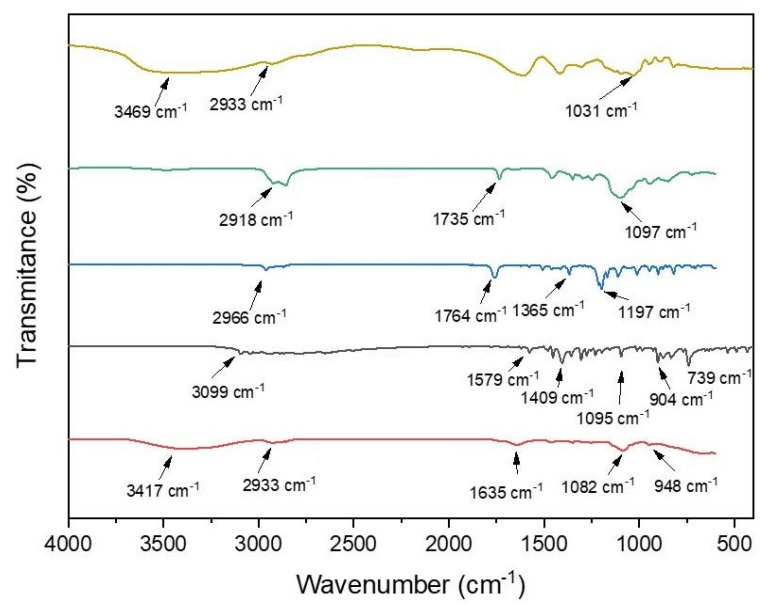
Fournier transform infrared spectroscopy (FTIR) spectra of sodium alginate, Tween 80, carvacryl acetate, thiabendazole, and emulsion with polymer matrix. Yellow line—sodium alginate; Green line—Tween; Blue line—carvacryl acetate; Gray line—thiabendazole; Red line—combination of carvacryl acetate, thiabendazole and sodium alginate.

Figure 1 depicts the FTIR spectra of ALG, Tween 80 (TW), TBZ, CA, and the emulsion CA/TBZ/ALG. ALG showed bands at approximately 1800 and 1620 cm^−1^ corresponding to COO- stretching and 1234 cm^−1^ attributed to COH stretching. TBZ showed absorption bands at 3099 cm^−1^ corresponding to CH stretching, absorption bands at 1579 cm^−1^ corresponding to NH stretching, and at 1409 cm^−1^ corresponding to C=C stretching of the aromatic ring. It also showed absorption bands at 1095 cm^−1^ corresponding to C-H folding in the plane and bands at 904 cm^−1^ and 739 cm^−1^ corresponding to CH folding outside the plane. TW showed a methyl group absorption band at 2918 cm^−1^, a band at approximately 2860 cm^−1^ due to -CH2 stretching, a band at 1735 cm^−1^ due to C=O, and a band at 1095 cm^−1^ corresponding to C-O-C stretching. CA showed bands at 2966 cm^−1^ corresponding to -CH2 stretching, and also at 1764 cm^−1^ (C=O stretching), 1365 cm^−1^ (C-O angular deformation), and 1197 cm^−1^. The CA/TBZ/ALG nanoemulsion exhibited overlapping bands with TW at 1097 cm^−1^, with ALG at 2933 cm^−1^, and with CA at 904 cm^−1^.

The CA/TBZ emulsion analyzed after 7 days showed a multimodal profile in size distribution (Supplementary Materials Figure S1A). Notably, the fraction of nanometric domains with an average size of 11 nm is smaller by approximately 30%. It is observed that most of the domains have an average size of 400 nm, with a wide range of sizes ranging between 100 and 1000 nm, and already present a fraction of particles with micrometric size, resulting from the agglomeration of smaller domains. CA/TBZ/ALG analyzed after 3 weeks of production showed a multimodal distribution (Supplementary Materials Figure S1B), where 86.5% of the emulsion is composed of nanometric domains, distributed as follows: 47% are sized droplets with an average particle size of 11 nm, 4.5% are composed of droplets with an average particle size of 100 nm, and 34% have domains with an average particle size of 480 nm. There is an occurrence of a peak containing 13.3% of the emulsion with micrometric domains, probably attributed to the agglomeration of the smaller domains due to the Oswald maturation process.

CA/LEV emulsions presented a larger particle size distribution with 53% of particles with a size of approximately 11 nm, 37% with sizes close to 500 nm, and a small fraction of particles (10%) with a size above 1000 nm, characterizing a nanoemulsion (Supplementary Materials Figure S2A). The CA/LEV/ALG emulsion presented larger particle sizes, with values very close to 1000 nm and was characterized as a microemulsion (Supplementary Materials Figure S2B).

**Figure 2:**
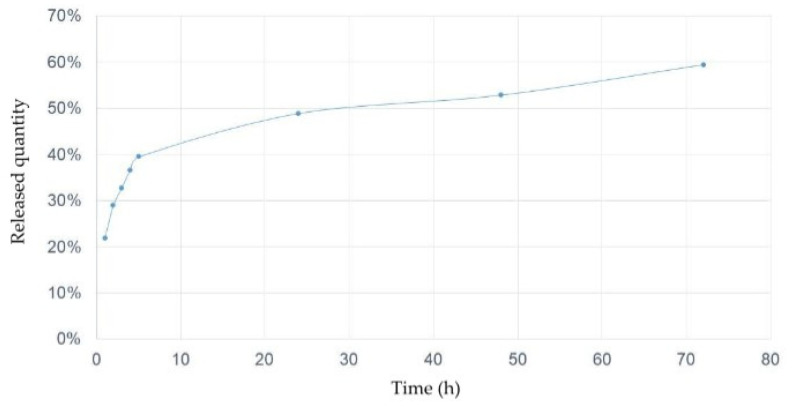
Release kinetics of the emulsion of carvacryl acetate and thiabendazole with sodium alginate (CA/TBZ/ALG).

The CA/TBZ/ALG release kinetics in the EHT time (48 h) where less than 60% of the active ingredients in the emulsion containing matrix were released (Figure 2).

Table 2 shows that the emulsions containing the ALG matrix (CA/TBZ/ALG and CA/LEV/ALG) present potentials with a charge of less than −55 mV, characterizing them as highly stable systems with a low tendency to agglomerate. On the other hand, emulsions produced without the matrix have an average potential of −20 mV, showing less stability, being more susceptible to Ostwald maturation, and forming larger agglomerates [15].

The concentrations of active constituents of formulations were determined, and the encapsulation efficiency of the emulsion CA/TBZ/ALG was 80.25 ± 5.69% and that of CA/LEV/ALG was 89.73 ± 3.42%. The obtained results showed that the two emulsions presented encapsulation efficiencies above 80%.

**Figure 3:**
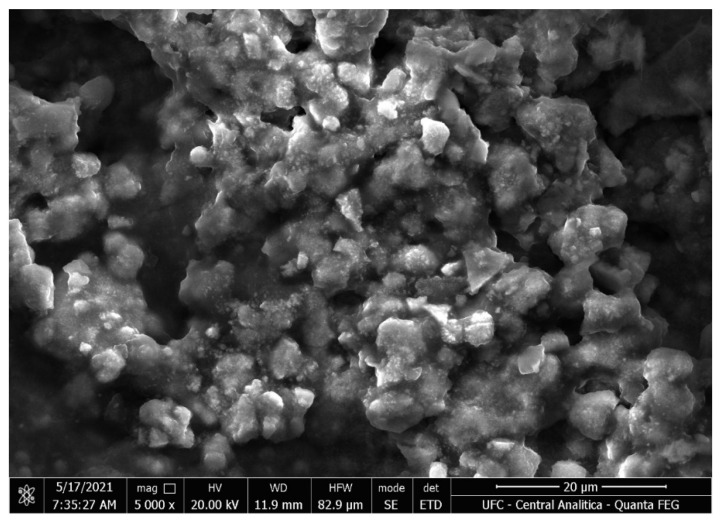
Scanning electron microscopy (SEM) image of nanoemulsion with thiabendazole and carvacryl acetate with a 1% sodium alginate matrix.

The morphology of the thiabendazole and carvacryl acetate nanoemulsion with a sodium alginate matrix visualized by SEM (Figure 3) presented spheroid particles with heterogeneous size. The nanoemulsion with thiabendazole and carvacryl acetate with a 1% sodium alginate had irregular morphology and aggregation among the particles. As already discussed by [27], these irregularities in shape are likely attributed to the drying process of the sample prior to SEM imaging. Larger microparticles formed by coalescence of particles with size range from 1–10 µm are shown, with nanoparticles adhered to the surface. These nanoparticles inclusions are nanodroplets, as already observed in other studies [15,28].

The effects of TBZ and CA using EHT on the anthelmintic-resistant *H. contortus* isolate are shown in Table 3. TBZ reduced the inhibition of egg hatching by 96.07% at a concentration of 0.05 mg/mL, while CA showed an inhibitory effect of 94.88% at a concentration of 8 mg/mL. Both had a dose-dependent effect, and the EC_50_ for TBZ was 0.011 mg/mL, while that for CA was 0.653 mg/mL (*p* < 0.05).

**Figure 4:**
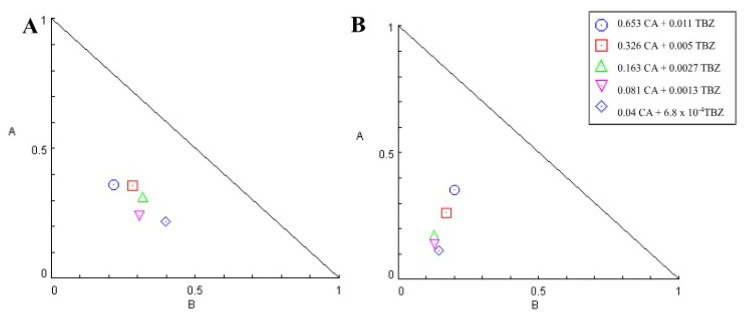
Isobologram of the thiabendazole and carvacryl acetate combination without (**A**) and with a matrix (**B**) in the inhibition of hatching of *Haemonchus contortus* eggs. Axes A and B correspond to carvacryl acetate and thiabendazole doses, respectively.

The effects of CA/TBZ and CA/TBZ/ALG were dose-dependent and are shown in Table 4. CA/TBZ had a mean CI of 0.55, and CA/TBZ/ALG had a mean CI of 0.36. At all analyzed concentrations, the CI of < 1 demonstrated synergism between the compounds. The SRs for CA/TBZ and CA/TBZ/ALG were 3.74 and 6.71, demonstrating a slight synergism in both cases. Figure 4 represents the isobologram formed by combination without (A) and with a matrix (B). The highest mean effect at concentrations of 0.011 mg/mL TBZ and 0.653 mg/mL CA was 86.48% in CA/TBZ and 87.53% in CA/TBZ/ALG.

The inhibitory effects of IVM, LEV, and CA on LDT using anthelmintic-resistant *H. contortus* isolates are shown in Table 5. IVM had an average effect equal to 100% at a concentration of 0.05 mg/mL, while LEV was 99.25% at a concentration of 0.8 mg/mL. CA had an effect of 100% at a concentration of 2 mg/mL, and all had a dose-dependent effect. The EC_50_ was 8.4 × 10^−4^ mg/mL for IVM, 145 × 10^−4^ mg/mL for LEV, and 383 × 10^−4^ mg/mL for CA (*p* < 0.05).

**Figure 5:**
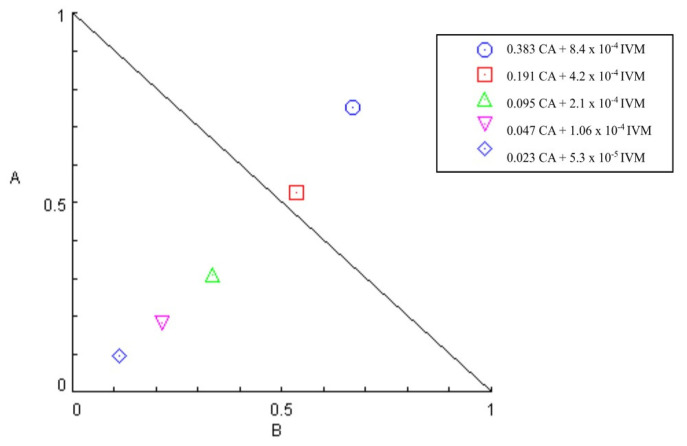
Isobologram of ivermectin and carvacryl acetate combination on *Haemonchus contortus* in larval development test. Axes A and B correspond to carvacryl acetate and ivermectin doses, respectively.

CA/IVM had a mean CI of 0.75, and the SR was 2.1. However, at the two highest concentrations, the CI was greater than 1, demonstrating antagonism (Table 6). The isobologram formed is shown in Figure 5.

**Figure 6:**
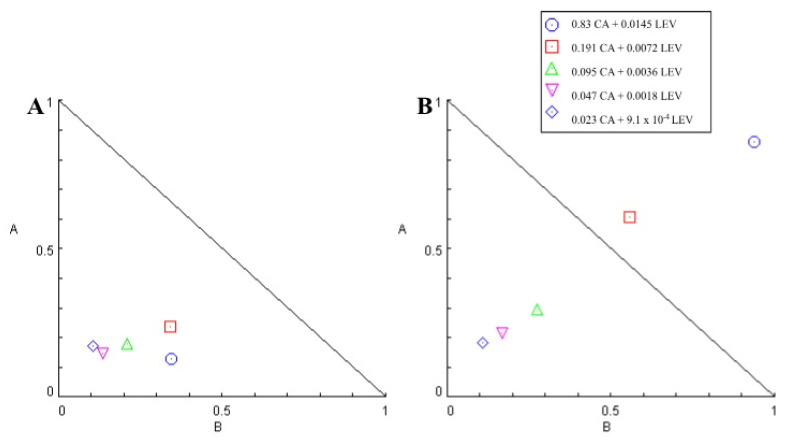
Isobologram of levamisole and carvacryl acetate combination without (**A**) and with matrix (**B**) on *Haemonchus contortus* in larval development test. Axes A and B correspond to carvacryl acetate and levamisole doses, respectively.

CA/LEV and CA/LEV/ALG had a dose-dependent effect. CA/LEV had a mean CI of 0.4, and CA/LEV/ALG had a mean CI of 0.93. CA/LEV had an SR of 5.51, and CA/LEV/ALG had an SR of 1.34. In all analyzed concentrations of CA/LEV, the CI was less than 1, demonstrating synergism between the compounds, and in CA/LEV/ALG, at the highest concentration, CI was > 1.5 and showed high antagonism (Table 7). The isobolograms formed by combinations without (A) and with a matrix (B) are shown in Figure 6.

**Figure 7:**
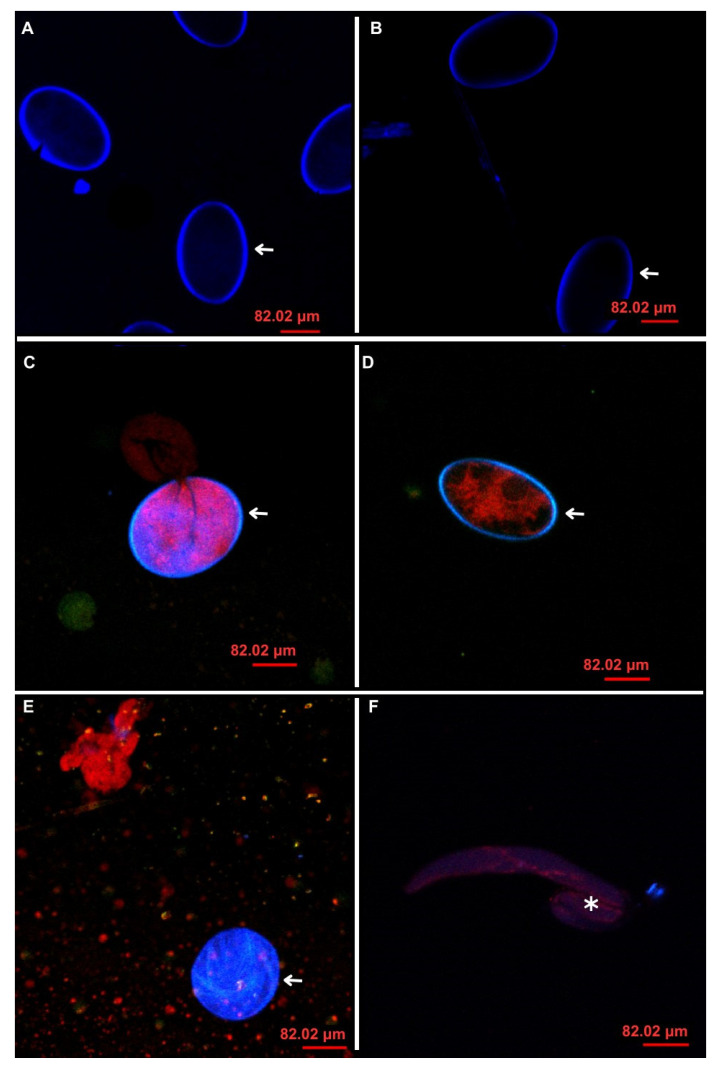
Confocal laser scanning microscopy images of *Haemonchus contortus* eggs: (**A**) Tween 3%; (**B**) 1% sodium alginate; (**C**) thiabendazole (0.05 mg/mL); (**D**) carvacryl acetate (8 mg/mL); (**E**) combination of thiabendazole and carvacryl acetate without matrix; and (**F**) combination of thiabendazole and carvacryl acetate with sodium alginate matrix (40× magnification). White arrows indicate an eggshell with or without larva, and asterisk shows a larva that is not fully developed.

Confocal laser scanning microscopy images of *H. contortus* eggs after treatment with TBZ, CA, and the CA/TBZ combination are shown in Figure 7. In the negative controls, treated with 3% Tween and 1% sodium alginate (A and B), only the eggshells stained with calcofluor were observed. In CA-treated eggs (D), larvae developed but were dead before fully hatching. In eggs treated with CA/TBZ (E), the development of larvae was not complete, egg breakage occurred, and shells wrinkled. In eggs treated with CA/TBZ/ALG (F), larvae were formed, but there was no hatching, and in the observed samples, the eggshells were not stained or were absent, so they were not visualized.

## 5. Discussion

The most used classes of anthelmintics in small ruminants are benzimidazoles (TBZ), imidazothiazoles (LEV), and macrocyclic lactones (IVM) [29]. TBZ was the first broad-spectrum, low-toxicity anthelmintic to be launched on the market [30]. Resistance to these classes is extremely common worldwide [31,32]. LEV is the most commonly used nicotinic agonist in small ruminants, and there are already several reports of anthelmintic resistance to this drug [17,18,33]. IVM is widely used in different species, including ruminants. CA, an acetylated derivative of carvacrol, is less toxic, and recent studies have proven its effect against *H. contortus*, the most important parasitic nematode of [8,34]. The combination of synthetic anthelmintics and compounds isolated from natural products to tackle resistance problems may provide new solutions for the control of GINs.

The EC_50_ of CA in the EHT (0.65 mg/mL) was lower than that found in previous studies (1.7 mg/mL) [8]. In the present study, a resistant *H. contortus* isolate was used, while in the study by [8], the *H. contortus* isolate was not characterized based on the status of anthelmintic sensitivity, and the effect of compounds varies depending on the resistance of the isolate used [35] and chemical substances [8,36,37]. In EHT, the highest concentration utilized of TBZ (0.05 mg/mL) inhibited 96.07% of egg hatching, demonstrating the high degree of resistance currently found toward this class of anthelmintics, while in a susceptible isolate, a dose of 0.1 μg/mL would have obtained a 99% ovicidal effect [20].

In EHT, the combination of CA and TBZ had a synergistic effect. In previous studies, the carvacrol/albendazole combination had an SR of 1.6 in EHT, demonstrating slight synergism, but without a significant difference in EC50 when compared to the anthelmintic used separately [38].

CA/TBZ/ALG showed a better effect than CA/TBZ (Table 3), a lower CI, and a higher SR, indicating a better synergism. However, there was no significant difference in the highest concentration used. The release kinetics also demonstrated that less than 60% of the active ingredient was released during the egg hatch period. This increase in efficacy may be linked to the fact that sodium alginate nanoparticles improve the potential carrier drugs and enhance their efficacy [39].

Several benzimidazole anthelmintics have been discontinued due to their ineffectiveness. The CA/TBZ combination may be a successful way of using benzimidazoles in small ruminants. Testing different surfactants, such as Tween 20, at different concentrations can improve the effectiveness of the CA/TBZ combination to develop a better formulation.

In LDT, the EC_50_ of CA (0.38 mg/mL) was similar to that found in previous studies (0.3 mg/mL) [8] even though different *H. contortus* isolates were used. IVM showed 0% inhibition of larval development at its lowest concentration (3 µg/mL), and in the standardized test by [21], the positive control used was 0.08 µg/mL with 95.2% inhibition of larval development, demonstrating that the isolated KOK is highly resistant to macrocyclic lactones.

CA had a synergistic effect with LEV in a free emulsion. The combination of carvacrol with LEV had an SR of 0.8 in the larval migration test, demonstrating mild synergism, without a significant difference in EC_50_ when compared to the anthelmintic used separately [38]. This demonstrates that CA possibly has carvacrol-like interactions when in combination with LEV. In both studies, the isolate used was resistant to LEV.

The synergism between CA and LEV was not expected because LEV acts as an agonist of nicotinic acetylcholine receptors (nAChRs), and carvacrol has an antagonist effect on these receptors [39,40,41,42]. However, the effect of the acetylated form has not yet been elucidated, probably being different from carvacrol.

The CA/IVM combination presented antagonism at the highest concentrations. If CA had a mechanism of action similar to carvacrol, it would probably have a synergistic effect, as carvacrol is a noncompetitive antagonist of nicotinic acetylcholine receptors, and thus, both would cause flaccid paralysis, potentiating the effect on the nematode [39,41,42].

The negative effect of the alginate matrix on the CA/LEV combination may be due to LEV being a polar compound that is soluble mainly in water [43] and CA being a nonpolar compound, and the addition of the matrix may have caused the retention of LEV in the aqueous solution, even though the formation of domains in macroscopy was not visible. The medium used in the LDT is solid and perhaps did not allow the release of compounds encapsulated by the sodium alginate matrix.

The sodium alginate matrix extended the shelf life of the CA/TBZ/ALG nanoemulsion; even 3 weeks after its production, it had a smaller particle size than the nanoemulsion CA/TBZ only 1 week after its production. The zeta potential of emulsions with sodium alginate showed less tendency to form agglomerates than formulations without a matrix, increasing the stability of the emulsions. In this case, the ALG external coating contributed to emulsion stabilization once it presented carboxylic groups with negative charges, preventing Oswald maturation due to the negative charges on the surface of the droplets. There are reports that corroborate these results, as ions can be absorbed on the surface of the droplet and promote an additional electrostatic barrier [44].

In confocal microscopy, the eggshell images were stained by calcofluor and visualized by blue color, and the propidium iodide of Live/Dead fluorescent dye binds to DNA dead cells in red. It was observed that in eggs treated with TBZ, there was no larval development (Figure 7C). TBZ inhibits tubulin polymerization and the consequent formation of microtubules, affecting mitosis and larval development [45]. The eggshell has an intermediate layer of chitin that is an association of chitinous fibrils surrounded by a layer of protein, preventing the action of CA even at the highest concentration. Larvae did develop; however, they did not hatch (Figure 7D). Apparently, the action of CA does not involve larval development but motility (Figure 7D). In CA/TBZ and CA/TBZ/ALG (Figure 7E,F), TBZ was at a lower concentration and was not able to inhibit larval development. CA probably acted after the larva hatched, causing paralysis and death, with red-stained larvae. The absence of the eggshell in the treatment with TBZ/CA/ALG may be caused by an effect of the ALG, which is disintegration, depending on the amount incorporated in the formulation. However, this effect was not observed in the group treated only with ALG; thus, the experiment needs to be reperformed for a better evaluation of the effect [46].

## 6. Conclusions

In conclusion, the combinations of CA with LEV and TBZ showed a synergistic interaction. The addition of the sodium alginate matrix in the CA/TBZ/ALG emulsion improved the effect on eggs. Furthermore, the emulsion with the sodium alginate matrix seems more stable than the emulsion without the matrix, as it presented a larger fraction of nanometric particles, with the second size fraction (400 nm) being the minority. In vitro experiments have shown that the association of commercial anthelmintics that are not effective against resistant nematodes when associated with CA can be effective. However, we must bear in mind that these results must be tested in vivo to obtain conclusive results.

## Figures and Tables

**Table 1:** Experimental conditions of nanoemulsion production with synthetic anthelmintic thiabendazole (TBZ), levamisole (LEV), and ivermectin (IVM) combined with carvacryl acetate (CA) with or without sodium alginate (ALG).

Sample Name	Oil Phase	Aqueous Phase
CA/TBZ	Tween 80/CA/TBZ	Distilled water
CA/TBZ/ALG	Tween 80/CA/TBZ	ALG 1%wt solution
CA/IVM	Tween 80/CA/IVM	Distilled water
CA/LEV	Tween 80/CA/LEV	Distilled water
CA/LEV/ALG	Tween 80/CA/LEV	ALG 1%wt solution
Control	Tween 80	Water

**Table 2:** Zeta potential of carvacryl acetate (CA) and anthelmintic emulsions with thiabendazole (TBZ) and levamisole (LEV) with and without alginate (ALG).

	Zeta Potential (ZP) ± Standard Deviation
CA/TBZ/ALG	−57.97mV ± 8.73
CA/TBZ	−26.20 mV ± 4.59
CA/LEV/ALG	−68.80 mV ± 2.36
CA/LEV	−21.60 mV ± 8.20

**Table 3:** Isolated effect (mean ± standard deviation) of thiabendazole and carvacryl acetate on the inhibition hatching of *Haemonchus contortus* eggs.

Thiabendazole (mg/mL)	Mean ± Standard Deviation	Carvacryl Acetate (mg/mL)	Mean ± Standard Deviation
0.05	96.07 ± 3.24 ^A^	8	94.88 ± 2.09 ^A^
2.5 × 10^−2^	76.61 ± 3.20 ^B^	4	90.76 ± 2.41 ^A^
1.25 × 10^−2^	52.77 ± 2.08 ^C^	2	83.42 ± 5.25 ^B^
6 × 10^−3^	28.80 ± 6.20 ^D^	1	60.04 ± 9.94 ^C^
3 × 10^−3^	14.18 ± 4.99 ^E^	0.5	37.68 ± 10.46 ^D^
Tween 3%	4.93 ± 1.18	Tween 3%	4.93 ± 1.18

Different capital letters indicate significantly different values in the column (*p* < 0.05).

**Table 4:** Effect (mean ± standard deviation) and combination index (CI) of the combination of thiabendazole (TBZ) and carvacryl acetate (CA) without (CA/TBZ) or with a 1% sodium alginate matrix (CA/TBZ/ALG) on the inhibition of hatching of *Haemonchus contortus* eggs.

CA (mg/mL)	TBZ (mg/mL)	CA/TBZ		CA/TBZ/ALG	
		Mean ± Standard Deviation	CI	Mean ± Standard Deviation	CI
0.65	1.1 × 10^−2^	86.47 ± 4.41 ^Aa^	0.59	87.53 ± 2.81 ^Aa^	0.55
0.33	5 × 10^−3^	65.82 ± 6.30 ^Ba^	0.65	77.93 ± 6.22 ^Bb^	0.43
0.16	2.7 × 10^−3^	41.66 ± 4.78 ^Ca^	0.63	67.33 ± 3.23 ^Cb^	0.31
0.08	1.3 × 10^−3^	28.42 ± 8.79 ^Da^	0.46	46.45 ± 6.09 ^Db^	0.27
0.04	6.8 × 10^−4^	12.71 ± 4.97 ^E^	0.45	24.08 ± 4.73 ^Eb^	0.26
Tween 3%	3.40 ± 2.01		4.59 ± 2.57		
Tween 3%	3.40 ± 2.01		4.59 ± 2.57		

Different capital letters indicate significantly different values in the column and lower in the row (*p* < 0.05).

**Table 5:** Effect (mean ± standard deviation) of ivermectin, levamisole, and carvacryl acetate on *Haemonchus contortus* in the larval development test.

Ivermectin (mg/mL)	Mean ± Standard Deviation	Levamisole (mg/mL)	Mean ± Standard Deviation	Carvacryl Acetate (mg/mL)	Mean ± Standard Deviation
0.05	100 ± 0.25 ^A^	0.8	99.25 ± 0.61 ^A^	2	100 ± 0 ^A^
2.5 × 10^−2^	97.74 ± 3.87 ^A^	0.4	84.43 ± 6.05 ^B^	1	97.49 ± 3.96 ^A^
1.25 × 10^−2^	68.06 ± 8.07 ^B^	0.2	63.81 ± 6.30 ^C^	0.5	46.97 ± 12.7 ^B^
6 × 10^−3^	44.03 ± 6.85 ^C^	0.1	35.34 ± 7.51 ^D^	0.25	29.27 ± 13.09 ^C^
3 × 10^−3^	0 ± 3.79 ^D^	0.05	5.92 ± 8.98 ^E^	0.13	0 ± 2.09 ^D^

Different capital letters indicate significantly different values in the column (*p* < 0.05).

**Table 6:** Effect (mean ± standard deviation) and combination index (CI) of the combination of ivermectin and carvacryl acetate on *Haemonchus contortus* in the larval development test.

CA (mg/mL)	IVM (mg/mL)	Mean ± Standard Deviation	CI
0.38	8.4 × 10^−4^	79.32 ± 7.48 ^A^	1.42
0.19	4.2 × 10^−4^	43.41 ± 7.78 ^B^	1.06
0.09	2.1 × 10^−4^	26.28 ± 14.9 ^C^	0.64
0.05	1.06 × 10^−4^	13.24 ± 14.48 ^C,D^	0.40
0.02	0.53 × 10^−4^	9.62 ± 10.68 ^D^	0.21

Different letters indicate significantly different values (*p* < 0.05).

**Table 7:** Effect (mean ± standard deviation) and combination index (CI) of levamisole (LEV) and carvacryl acetate (CA) combinations without and with sodium alginate matrix (ALG) on *Haemonchus contortus* in larval development test.

CA (mg/mL)	LEV (mg/mL)	CA/LEV		CA/LEV/ALG	
		Mean ± Standard Deviation	CI	Mean ± Standard Deviation	CI
0.38	145 × 10^−4^	99.33 ± 0.54 ^Aa^	0.47	57.0 ± 10.54 ^Ab^	1.80
0.19	72 × 10^−4^	85.35 ± 5.41 ^Ba^	0.58	36.4 ± 7.56 ^Bb^	1.16
0.09	36 × 10^−4^	64.77 ± 5.38 ^Ca^	0.39	37.9 ± 4.93 ^Bb^	0.57
0.05	18 × 10^−4^	37.01 ± 8.61 ^Da^	0.28	18.4 ± 7.47 ^Cb^	0.38
0.02	9.06 × 10^−4^	5.83 ± 6.31 ^Ea^	0.28	5.05 ± 6.10 ^C,Da^	0.29
ALG 1%				0.27 ± 0.55 ^D^	

Different capital letters indicate significantly different values in the column and lower in the row (*p* < 0.05).

## Data Availability

The data presented in this study are available on request from the corresponding author.

## Supplementary Materials

The following supporting information can be downloaded at: https://www.mdpi.com/article/10.3390/ani14071007/s1, Figure S1: Particle size of thiabendazole and carvacryl acetate emulsion without matrix (A) and with matrix (B); Figure S2: Particle size of levamisole and carvacryl acetate emulsion without matrix (A) and with matrix (B).

## References

[1] Selzer P.M., Epe C. (2020). Antiparasitics in animal health: Quo Vadis?. Trends Parasitol..

[2] Bartram D.J., Leathwick D.M., Taylor M.A., Geurden T., Maeder S.J. (2012). The role of combination anthelmintic formulations in the sustainable control of sheep nematodes. Vet. Parasitol..

[3] Geary T.G., Hosking B.C., Skuce P.J., Von Samson-Himmelstjerna G., Maeder S., Holdsworth P., Pomroy W., Vercruysse J. (2012). World Association for the Advancement of Veterinary Parasitology (WAAVP) Guideline: Anthelmintic combination products targeting nematode infections of ruminants and horses. Vet. Parasitol..

[4] Wang H., Huang Y. (2020). Combination therapy based on nano codelivery for overcoming cancer drug resistance. Med. Drug Discov..

[5] Anthony J.P., Fyfe L., Smith H. (2005). Plant active components–a resource for antiparasitic agents?. Trends Parasitol..

[6] Lanusse C., Canton C., Virkel G., Alvarez L., Costa-Junior L., Lifschitz A. (2018). Strategies to optimize the efficacy of anthelmintic drugs in ruminants. Trends Parasitol..

[7] Costa-Junior L., Silva C.R., Macedo S.R.D., Campos N.R.C.L., Lifsctiz A. Association of synthetic anthelmintics and natural monoterpenes against *Haemonchus contortus*. Proceedings of the 26th International Conference of the World Association for the Advancement of Veterinary Parasitology.

[8] Andre W.P.P., Ribeiro W.L.C., Cavalcante G.S., Santos J.M.L., Macedo I.T.F., De Paula H.C.B., De Freitas R.M., De Morais S.M., De Melo J.V., Bevilaqua C.M.L. (2016). Comparative efficacy and toxic effects of carvacryl acetate and carvacrol on sheep gastrointestinal nematodes and mice. Vet. Parasitol..

[9] Jaiswal M., Dudhe R., Sharma P.K. (2004). Nanoemulsion: An advanced mode of drug delivery system. Biotech.

[10] Tadros T., Izquierdo P., Esquena J., Solans C. (2004). Formation and stability of nanoemulsions. Adv. Colloid. Interface Sci..

[11] Bouchemal K., Briançon S., Perrier E., Fessi H. (2004). Nano-emulsion formulation using spontaneous emulsification: Solvent, oil and surfactant optimisation. Int. J. Pharm..

[12] Campolo O., Giunti G., Laigle M., Michel T., Palmeri V. (2020). Essential oil-based nano-emulsions: Effect of different surfactants, sonication and plant species on physicochemical characteristics. Ind. Crop. Prod..

[13] Severino P., da Silva C.F., Andrade L.N., de Lima Oliveira D., Campos J., Souto E.B. (2019). Alginate nanoparticles for drug delivery and targeting. Curr. Pharm. Des..

[14] Matos F.J.A. (2009). Introdução a Fitoquímica Experimental.

[15] Abreu F.O.M.S., Costa E.F., Cardial M.R.L., André W.P.P. (2020). Polymeric nanoemulsions enriched with *Eucalyptus citriodora* essential oil. Polímeros.

[16] Park S.J., Jeong U.H., Lee J.W., Park J.S. (2010). Preparation and characterization of bovine serum albumin-loaded cationic liposomes: Effect of hydration phase. J. Pharm. Investig..

[17] Neveu C., Charvet C., Fauvin A., Cortet J., Castagnone-Sereno P., Cabaret J. (2007). Identification of levamisole resistance markers in the parasitic nematode *Haemonchus contortus* using a cDNA-AFLP approach. Parasitology.

[18] Fauvin A., Charvet C., Issouf M., Cortet J., Neveu C. (2010). cDNA-AFLP analysis in levamisole-resistant *Haemonchus contortus* reveals alternative splicing in a nicotinic acetylcholine receptor subunit. Mol. Biochem. Parasitol..

[19] Hubert J., Kerboeuf D.A. (1992). Microlarval development assay for the detection of anthelmintic resistance in sheep nematodes. Vet. Rec..

[20] Coles G.C., Jackson F., Pomroy W.E., Prichard R.K., Von Samson-Himmelstjerna G., Silvestre A., Taylor M.A., Vercruysse J. (2006). The detection of anthelmintic resistance in nematodes of veterinary importance. Vet. Parasitol..

[21] Camurça-Vasconcelos A.L.F., Bevilaqua C.M.L., Morais S.M., Maciel M.V., Costa C.T.C., Macedo I.T.F., Oliveira M.L.B., Braga R.R., Silva R.A., Vieira L.S. (2007). Anthelmintic activity of *Croton zehntneri* and *Lippia sidoides* essential oils. Vet. Parasitol..

[22] Roberts F.H.S., O’Sullivan P.J. (1950). Methods for egg counts and larval cultures for strongyles infesting the gastro-intestinal tract of cattle. Aust. J. Agric. Res..

[23] Castelo-Branco D.S.C.M., Riello G.B., Vasconcelos D.C., Guedes G.M.M., Serpa R., Bandeira T.J.P.G., Monteiro A.J., Cordeiro R.A., Rocha M.F.G., Sidrim J.J.C. (2016). Farnesol increases the susceptibility of *Burkholderia pseudomallei* biofilm to antimicrobials used to treat melioidosis. J. Appl. Microbiol..

[24] Chou T.C., Martin N. (2005). CompuSyn for Drug Combinations: PC Software and User’s Guide: A Computer Program for Quantitation of Synergism and Antagonism in Drug Combinations, and the Determination of IC50 and ED50 and LD50 Values.

[25] Chou T.C. (2006). Theoretical basis, experimental design, and computerized simulation of synergism and antagonism in drug combination studies. Pharmacol. Rev..

[26] Bielza P., Espinosa P.J., Quinto V., Abellan J., Contreras J. (2007). Synergism s tudies with binary mixtures of pyrethroid, carbamate and organophosphate insecticides on Frankliniella occidentalis (Pergande). Pest. Manag. Sci..

[27] Jawaid T., Alaseem A.M., Khan M.M., Mukhtar B., Kamal M., Anwer R., Ahmed S., Alam A. (2023). Preparation and evaluation of nanoemulsion of Citronella essential oil with improved antimicrobial and anti-Cancer properties. Antibiotics.

[28] Bouriche S., Alonso García Á., Cárceles-Rodríguez C., Rezgui F., Fernández-Varón E. (2021). An in vivo pharmacokinetic study of metformin microparticles as an oral sustained release formulation in rabbits. BMC Vet. Res..

[29] Nixon S.A., Saez N.J., Herzig V., King G.F., Kotze A.C. (2019). The antitrypanosomal diarylamidines, diminazene and pentamidine, show anthelmintic activity against *Haemonchus contortus* in vitro. Vet. Parasitol..

[30] Křížová-Forstová V., Lamka J., Cvilink V., Hanušová V., Skálová L. (2011). Factors affecting pharmacokinetics of benzimidazole anthelmintics in food-producing animals: The consequences and potential risks. Res. Vet. Sci..

[31] Lambert S.M., Nishi S.M., Mendonça L.R., Souza B.M.P., Julião F., Gusmão P., Almeida M.A.O. (2017). Genotypic profile of benzimidazole resistance associated with SNP F167Y and F200Y beta-tubulin gene in Brazilian populations of *Haemonchus contortus* of goats. Vet. Parasitol. Reg. Stud. Rep..

[32] Santos J.M.L., Monteiro J.P., Ribeiro W.L.C., Macedo I.T.F., Araújo Filho J.V., Andre W.P.P., Araùjo P.R.M., Vasconcelos J.F., Freitas E.P., Camurça-Vasconcelos A.L.F. (2017). High levels of benzimidazole resistance and β-tubulin isotype 1 SNP F167Y in *Haemonchus contortus* populations from Ceará State, Brazil. Small Rum. Res..

[33] Santos J.M.L., Vasconcelos J.F., Frota G.A., Freitas E.P., Teixeira M., Silva L.V., Bevilaqua C.M.L., Monteiro J.P. (2019). Quantitative molecular diagnosis of levamisole resistance in populations of *Haemonchus contortus*. Exp. Parasitol..

[34] Moraes J., Carvalho A.A.L., Nakano E., Almeida A.A.C., Marques T.H.C., Andrade L.N., Freitas M., Sousa D.P. (2013). Anthelmintic activity of carvacryl acetate against *Schistosoma mansoni*. Parasitol. Res..

[35] Calderón-Quintal J.A., Torres-Acosta J.F.J., Sandoval-Castro C.A., Alonso M.A., Hoste H., Aguilar-Caballero A. (2010). Adaptation of *Haemonchus contortus* to condensed tannins: Can it be possible?. Arch. Med. Vet..

[36] Mansfield L.S., Gamble H.R., Fetterer R.H. (1992). Characterization of the eggshell of *Haemonchus contortus*: I structural components. Comp. Biochem. Physiol..

[37] Wharton D.A. (1980). Nematode egg-shells. Parasitology.

[38] Silva C.R., Lifschitz A.L., Macedo S.R., Campos N.R., Viana-Filho M., Alcântara A.C., Araujo J.G., Alencar L.M.R., Costa-Junior L.M. (2021). Combination of synthetic anthelmintics and monoterpenes: Assessment of efficacy, and ultrastructural and biophysical properties of *Haemonchus contortus* using atomic force microscopy. Vet. Parasitol..

[39] Ryan M.F., Byrne O. (1988). Plant-insect coevolution and inhibition of acetylcholinesterase. J. Chem. Ecol..

[40] Moreno-Guzmán M.J., Coles G.C., Jiménez-González A., Criado-Fornelio A., Ros-Moreno R.M., Rodríguez-Caabeiro F. (1998). Levamisole binding sites in *Haemonchus contortus*. Int. J. Parasitol..

[41] Mottier L., Lanusse C. (2001). Bases moleculares de la resistência a fármacos. Rev. Med. Vet..

[42] Marjanovic D.S., Zdravković N., Milovanović M., Trailović J.N., Robertson A.P., Todorović Z., Trailović S.M. (2020). Carvacrol acts as a potent selective antagonist of different types of nicotinic acetylcholine receptors and enhances the effect of monepantel in the parasitic nematode *Ascaris Suum*. Vet. Parasitol..

[43] Zhang P., Wan Y., Zhang C., Zhao R., Sha J., Li Y., Li T., Ren B. (2019). Solubility and mixing thermodynamic properties of levamisole hydrochloride in twelve pure solvents at various temperatures. J. Chem. Thermodyn..

[44] Santana R.C., Perrechil F.A., Cunha R.L. (2013). High-and low-energy emulsifications for food applications: A focus on process parameters. Food Eng. Rev..

[45] Lacey E. (1988). The role of the cytoskeletal protein, tubulin, in the mode of action and mechanism of drug resistance to benzimidazoles. Int. J. Parasitol..

[46] Tønnesen H.H., Karlsen J. (2002). Alginate in drug delivery systems. Drug Dev. Ind. Pharm..

