# Supplementary material for: Effect of the Combination of Synthetic Anthelmintics with Carvacryl Acetate in Emulsions with and without a Sodium Alginate Matrix on Haemonchus contortus
Source: Animals (Basel). 2024 Mar 26;14(7):1007. doi: 10.3390/ani14071007 (PMC11011019; doi:10.3390/ani14071007)
Supplement: Supplementary file 1 [file animals-14-01007-s001.zip › animals-2899881-supplementary.pdf]

Supplementary material

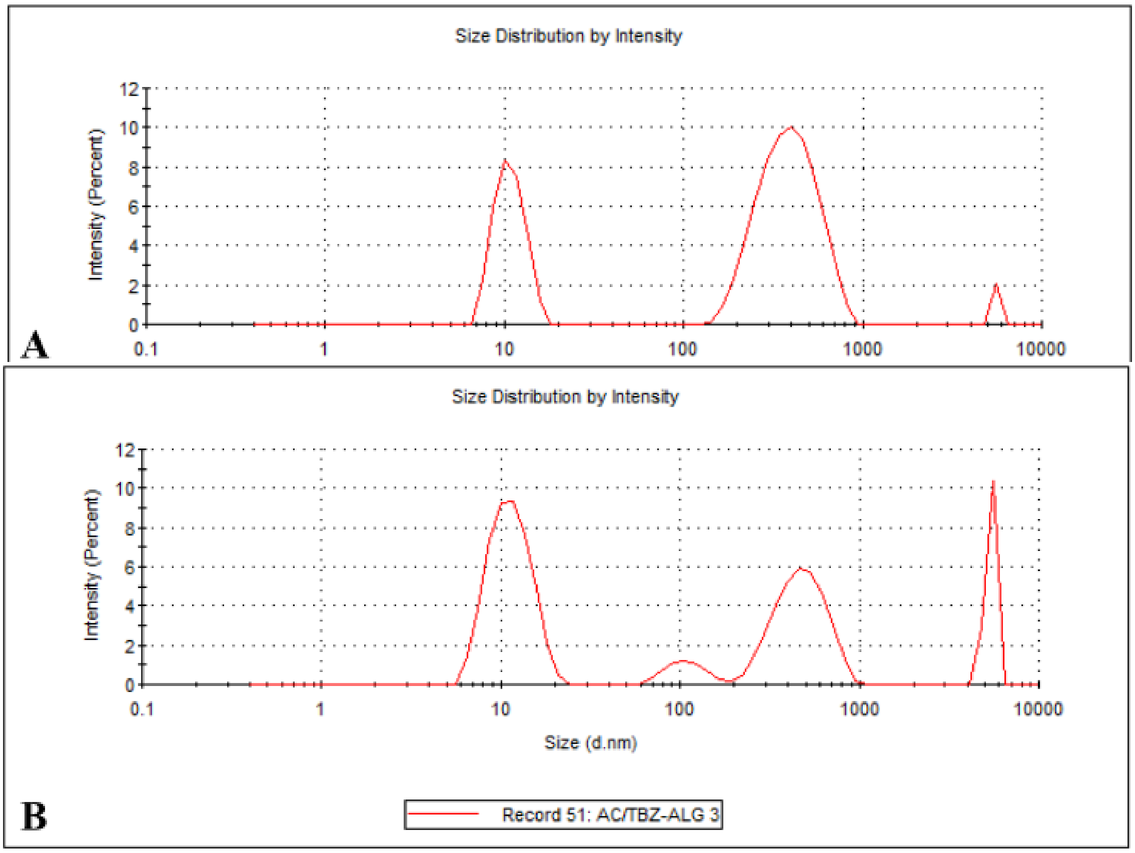

Figure S1: Particle size of thiabendazole and carvacryl acetate emulsion without matrix (A) and with matrix (B).

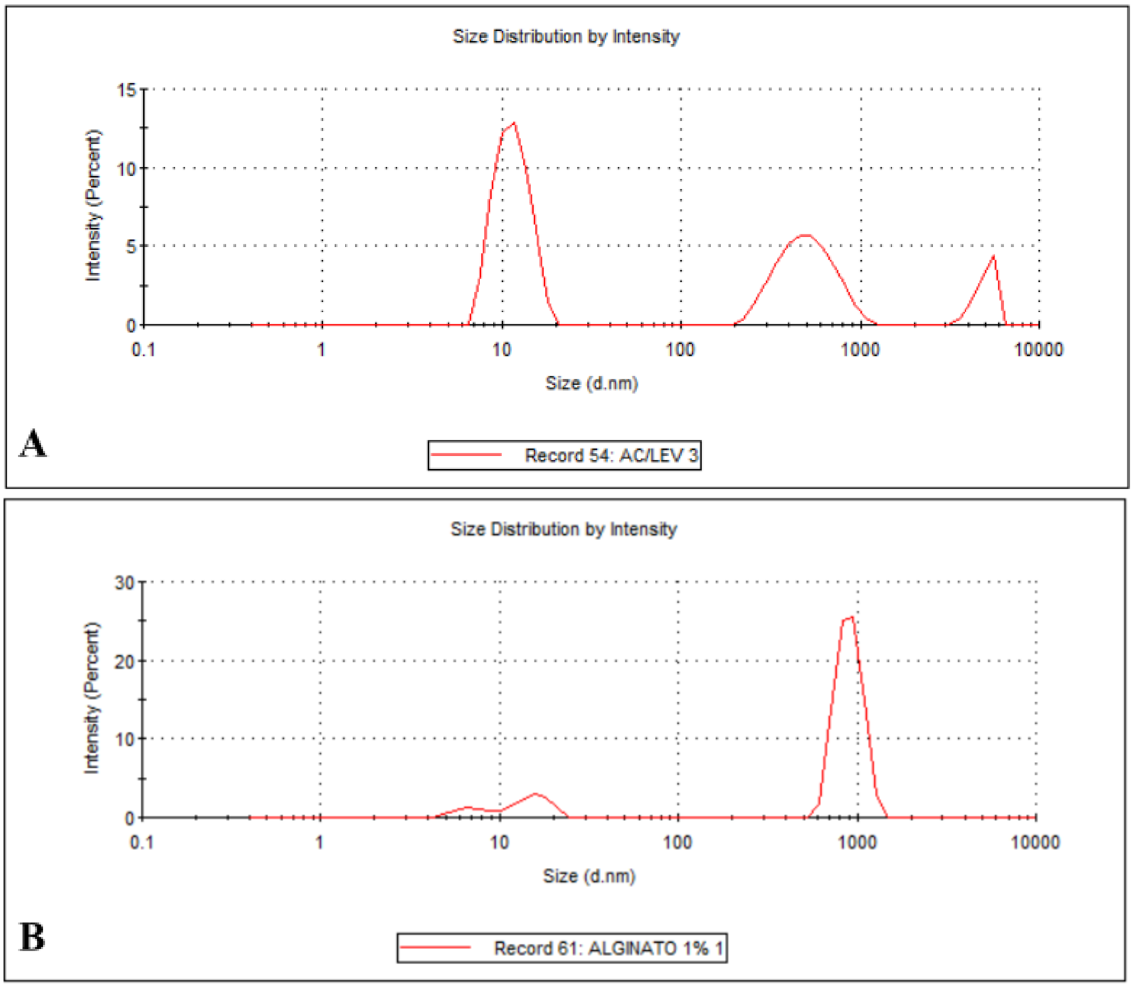

Figure S2: Particle size of levamisole and carvacryl acetate emulsion without matrix (A) and with matrix (B).
